# Tubuloreticular inclusions in juvenile dermatomyositis: a diagnostically useful marker?

**DOI:** 10.1186/1546-0096-12-S1-P91

**Published:** 2014-09-17

**Authors:** Shireena A Yasin, Erdal Sag, Katie Arnold, Glenn Anderson, Clarissa Pilkington, Simon ML Paine, Janice L Holton, Lucy R Wedderburn, Thomas S Jacques

**Affiliations:** 1 Infection, Inflammation and Rheumatology Section, Institute of Child Health, UCL, London, UK; 2 Histopathology, Great Ormond Street Hospital, London, UK; 3 Rheumatology, Great Ormond Street Hospital, London, UK; 4 Birth Defects Research Centre, Institute of Child Health, UCL, London, UK; 5 Molecular Neuroscience, MRC centre for neuromuscular diseases, Institute of Neurology, UCL, London, UK

## Introduction

Juvenile Dermatomyositis (JDM) is a rare life threatening disease affecting children. Symptoms include severe proximal muscle weakness and characteristic skin rashes. Vascular pathology is often a key finding in JDM including typical features of capillary drop out and abnormal blood vessel endothelial cells. We have observed that another common finding in JDM biopsies is the presence of tubuloreticular inclusions (TRI) detected by electron microscopy (EM) in blood vessel endothelial cells in muscle and the overlying skin. These are tubule-like structures within cisternae of endoplasmic reticulum.

## Objectives

The aim of this study was to determine the frequency and specificity of TRIs in JDM biopsies compared to muscle biopsies investigated for other pathologies

## Methods

The UK JDM Biomarker and Cohort Study (JDBCS) provides access to a large JDM cohort, with linked samples and biopsies, (n=446, biopsies n=135). We examined pathology by EM of 41 JDM biopsies and recorded reports of TRIs in blood vessel endothelial cells.

## Results

Tubuloreticular inclusions were demonstrated in blood vessel endothelial cells in 80% of JDM muscle biopsies (33/41) and in the overlying clinically unaffected skin in 78% of cases (26/33) where this tissue was available. In contrast no TRIs in vessel endothelial cells had previously been reported in muscle biopsies investigated for other pathologies in children (n>500).

## Conclusion

The high number of JDM muscle biopsies with identified TRIs compared to control biopsies investigated for other diseases suggests that TRI’s are a specific marker of JDM pathology. TRIs have also been found in other diseases such as glomerulonephritis (GN), lupus nephritis (LN), HIV and Degos disease and are suggested to be a biomarker of type1 interferon (IFN) exposure. Type 1 IFN gene and chemokine signature is thought to be associated with JDM pathology and clinical features. The high specificity of TRIs in vessel endothelium in JDM biopsies compared to non-JDM biopsies suggests that their detection is useful in supporting a diagnosis of JDM in patients.

## Disclosure of interest

None declared.

